# Transcatheter Mitral Valve Intervention: Current and Future Role of Multimodality Imaging for Device Selection and Periprocedural Guidance

**DOI:** 10.3390/medicina60071082

**Published:** 2024-07-01

**Authors:** Leonardo Brugiatelli, Marco Rolando, Carla Lofiego, Marco Fogante, Irene Capodaglio, Francesca Patani, Paolo Tofoni, Kevin Maurizi, Marco Nazziconi, Arianna Massari, Giulia Furlani, Giovanni Signore, Paolo Esposto Pirani, Nicolò Schicchi, Antonio Dello Russo, Marco Di Eusanio, Fabio Vagnarelli

**Affiliations:** 1 “G.M. Lancisi” Cardiovascular Center, 60126 Ancona, Italy; 2 Department of Biomedical Sciences and Public Health, Marche Polytechnic University, 60121 Ancona, Italy; 3 Maternal-Child, Senological, Cardiological Radiology and Outpatient Ultrasound, Department of Radiological Sciences, University Hospital of Marche, 60126 Ancona, Italy; 4 Department of Transalational Medical Sciences, University of Campania “Luigi Vanvitelli”, 80131 Naples, Italy; 5 Cardiovascular Radiological Diagnostics, Department of Radiological Sciences, University Hospital of Marche, 60126 Ancona, Italy; 6 Cardiac Surgery Department, Polytechnic University of Marche, AOU delle Marche, 60121 Ancona, Italy

**Keywords:** TMVI, MR, pre-procedural planning, intra-procedural guidance, multi-modality imaging

## Abstract

Mitral regurgitation (MR) is a broadly diffuse valvular heart disease (VHD) with a significant impact on the healthcare system and patient prognosis. Transcatheter mitral valve interventions (TMVI) are now well-established techniques included in the therapeutic armamentarium for managing patients with mitral regurgitation, either primary or functional MR. Even if the guidelines give indications regarding the correct management of this VHD, the wide heterogeneity of patients’ clinical backgrounds and valvular and heart anatomies make each patient a unique case, in which the appropriate device’s selection requires a multimodal imaging evaluation and a multidisciplinary discussion. Proper pre-procedural evaluation plays a pivotal role in judging the feasibility of TMVI, while a cooperative work between imagers and interventionalist is also crucial for procedural success. This manuscript aims to provide an exhaustive overview of the main parameters that need to be evaluated for appropriate device selection, pre-procedural planning, intra-procedural guidance and post-operative assessment in the setting of TMVI. In addition, it tries to give some insights about future perspectives for structural cardiovascular imaging.

## 1. Introduction

**Figure 1:**
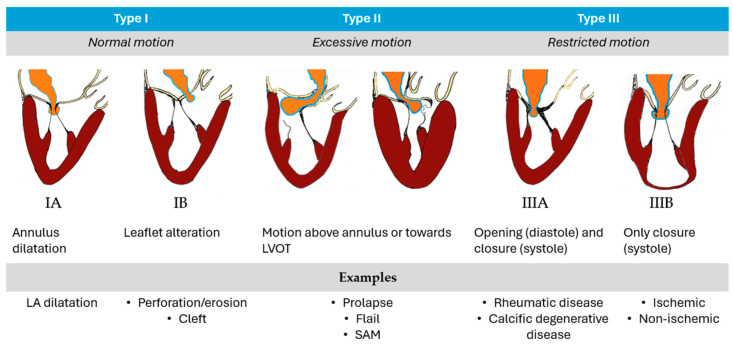
Carpentier’s classification of MR. Ischemic: papillary muscle displacement/dysfunction, asynchrony. Non-ischemic: dilated cardiomyopathy, asynchrony. LVOT, left ventricular outflow tract; SAM, systolic anterior movement.

Mitral regurgitation (MR) represents the second most prevalent valve disease in Europe, with a prevalence that increases with age and a reduced overall survival, if left untreated [1,2,3]. Etiologically, MR is classified as primary or organic (PMR) and secondary or functional (FMR). In PMR, the primum movens is an intrinsic leaflet pathology, whereas in FMR the disease arises from ventricular remodeling/dysfunction (V-FMR) or from left atrial dilatation (A-FMR), and the leaflets undergo further secondary adaptation [4]. The Carpentier classification is most often used to emphasize the specific mechanism of MR (Figure 1).

This classification holds therapeutic implications and was originally used to guide surgical strategy. Type I MR may require annuloplasty in cases of annular dilatation, or pericardial patch in cases of leaflet perforation. In Type II MR, different techniques (chordal implantation, chordal transfer, leaflet resection, sliding plasty, edge-to-edge), usually associated to annuloplasty, may be used. In Type IIIa, surgical options include commissurotomy, chordal fenestration, or replacement. In Type IIIb, the surgical approach consists of annuloplasty in most cases. The transcatheter approach may be the leaflet/chordal/annular approach or transcatheter valve replacement depending on the anatomic assessment.

In recent years, particular attention has been paid to FMR, associated with worse outcomes than PMR, clarifying the existence of two different forms with distinct natures and prognoses [5,6]:

▪ **A-FMR.** The main mechanism is represented by annular dysfunction or dilatation and atriogenic leaflet tethering with reduced leaflet remodeling, leading to an annulus–leaflet area imbalance. It usually involves patients with preserved left ventricular (LV) systolic function, particularly those suffering from atrial fibrillation (AF) [7,8]. Optimal treatment remains debated and includes rhythm control and different transcatheter and surgical procedures, with the latter potentially able to treat all the mechanisms of the disease: plication for left atrium (LA) enlargement, annuloplasty for annulus dilatation, patch augmentation for insufficient leaflet remodeling, and the Cox–Maze procedure for AF [9,10]. When feasible, MVr is probably associated with a better outcome than MVR [11].
▪ **V-FMR.** The main mechanism is LV dilatation and/or systolic dysfunction with global or regional remodeling of LV and/or asynchrony, leading to symmetric or asymmetric tethering of mitral leaflets. The best treatment usually involves a comprehensive use of the “classical” heart failure management strategies: optimal medical treatment, myocardial revascularization if indicated, cardiac resynchronization therapy, and transcatheter edge-to-edge repair (TEER) [12].

The arrival of transcatheter approaches, in terms of both repair (TMVr) and replacement (TMVR), has offered an alternative to surgery in the spectrum of patients with MR, particularly for patients deemed unsuitable or at an elevated risk for surgical procedures [13]. The purpose of this review is to summarize the current literature on transcatheter mitral valve intervention (TMVI), highlighting the importance of echocardiography and multimodal imaging from the pre-procedural planning to the intra-procedural guidance and the post-procedural evaluation. Furthermore, relevant insights in the field of emerging imaging techniques will be addressed.

## 2. Materials and Methods

A comprehensive review of the literature was performed by querying PubMed, Embase, Cochrane with the following keywords: “transcatheter mitral valve repair”, “transcatheter mitral valve replacement”, “TMVR”, “TMVr”, “transcatheter edge-to-edge repair”, ”MitraClip”, “PASCAL”, “Tendyne”, “primary mitral regurgitation”, “secondary mitral regurgitation”, “Carillon”, “Cardioband”, “multimodality imaging in structural heart disease”, and “transesophageal guidance in structural heart intervention”. Studies that contained relevant information were selected and included in the subsequent discussion.

## 3. Transcatheter Mitral Valve Intervention

**Figure 2:**
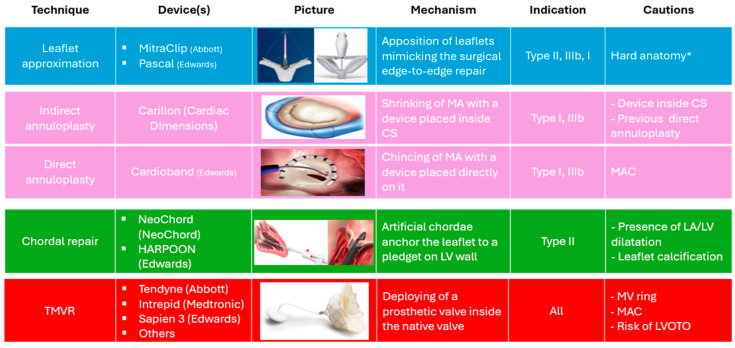
Currently approved device for TMVI. Type I, II, and III refer to the Carpentier classifications of mitral regurgitation. MA, mitral annulus; CS, coronary sinus; MAC, mitral annulus calcification; LA, left atrium; LV, left ventricle; TMVR, transcatheter mitral valve replacement; LVOTO, left ventricular outflow tract obstruction. * For a more detailed discussion of the challenging anatomic criteria refer to the Section 4.1.

TMVr procedure encompasses the use of the following different devices and conceptually different techniques (Figure 2):

- *Leaflet’s approximation:* MitraClip (Abbott Cardiovascular, Plymouth, MN, USA), PASCAL (Edwards Lifesciences, Irvine, CA, USA);
- *Direct annuloplasty:* Cardioband (Edwards Lifesciences, Irvine, CA, USA);
- *Indirect annuloplasty:* Carillon Mitral Contour System (Cardiac Dimensions, Washington, DC, USA);
- *Chordal repair:* HARPOON (Edwards Lifesciences, Irvine, CA, USA). NeoChord (NeoChord Inc. Louise Park, MN, USA).

TMVR is now represented exclusively by the Tendyne system (Abbott Cardiovascular, Plymouth, MN, USA), the only one with CE mark approval, whereas other devices are still under evaluation in clinical studies.

**Leaflet approximation.** TEER is the most widely used and studied TMVI and is inspired by Alfieri’s surgical edge-to-edge suture between the free edge of the anterior (AL) and posterior mitral leaflet (PL). The two devices available are the MitraClip and the PASCAL system, both of which are implantable by venous transfemoral access, trans-septal puncture (TSP), and general anesthesia with transesophageal (TEE) guidance.

**Figure 3:**
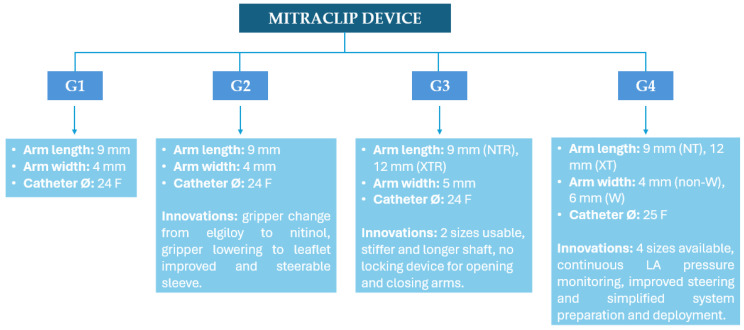
Evolution of the MitraClip System through different device generations.

The MitraClip system received CE mark approval in 2008 and was subsequently upgraded up to the last generation device (Figure 3).

It has a cobalt–chromium and nitinol core with a polyester cover, designed to promote tissue growth. Two arms and grippers allow the device to close in on itself, capturing and approximating the leaflets. The system is formed by a steerable guide catheter (SGC) and a clip delivery system (CDS). The SGC consists of a catheter with an echogenic tapered dilator. The CDS is introduced into the body through the SGC and is composed of (a) the delivery catheter, (b) the steerable sleeve, and (c) the MitraClip device. The device was firstly tested in the EVEREST I trial, showing the good efficacy and safety of the procedure both in PMR and FMR, and afterwards also in the EVEREST II trial, where it was associated with superior safety and similar improvement of clinical outcomes, but was less effective at reducing MR than conventional surgery [14,15]. Subsequently, attention shifted towards FMR, which had been underrepresented in earlier trials.

In this context, the MITRA-FR and COAPT trials have contributed to better delineating the subpopulation of FMR that may benefit the most from the procedure (the so called “COAPT-like” phenotype). This distinction holds true even when considering long-term outcomes [16,17]. The practical conclusion is that the MitraClip device yields the best result in patients with more severe MR and less advanced LV disease (characterized as “disproportionate MR”). Conversely, in patients with more advanced LV disease and less severe MR, known as “proportionate MR”, the procedure does not appear to affect prognosis.

Moreover, the clinical benefit of TEER may be hampered by several factors, despite optimal procedural success in reducing MR. These factors include severe tricuspid regurgitation (TR), severe pulmonary hypertension, and right ventricular dysfunction, which should be carefully considered during patients’ selection [18,19,20].

The PASCAL system was designed to overcome some of the limitations associated with early-generation MitraClip devices, including limited maneuverability within the LA, limited implant dimensions, risk of leaflet damage in case of multiple grasping attempts, and the potential for chordal injury in complex lesions, particularly in commissural zones. It consists of two components: an implant system (IS) and a guide sheath (GS). The IS consists of the steerable catheter (outermost layer), the implant catheter (innermost layer), and the implant device on its distal end; the implant is made of nitinol and is formed by (1) a central spacer, (2) two juxtaposed paddles, and (3) two different clasps; the spacer covers the regurgitant orifice area (ROA) and provides a surface for leaflets’ coaptation, while the paddles facilitate the leaflets’ approximation and the clasps adhere on the leaflets’ atrial side with only a row of grippers (4–6 rows in MitraClip system). The GS set includes a steerable guide sheath and an introducer. One of the main features of the system is its atraumatic behavior towards the leaflets. This is achieved through the shape and width of the paddles, which allow for more homogeneous stress and tension release on the leaflets. Additionally, the elongation properties of the device enable safe retraction from the sub-valvular area. Another significant advantage is the possibility of independent grasping, a feature that is only available in the fourth generation MitraClip system. In 2020, a second smaller device, the PASCAL Ace, was introduced to the market, further expanding the system’s compatibility with different valve anatomies. The procedure for PASCAL implantation is similar to that of the MitraClip and is performed under general anesthesia, with TEE guidance and venous femoral access using transseptal puncture (TSP). The PASCAL system received CE approval for both primary mitral regurgitation (PMR) and functional mitral regurgitation (FMR) after demonstrating good safety, feasibility, and long-term durability in the CLASP study [21].

*MitraClip vs. PASCAL.* The CLASP IID/IIF (ClinicalTrials.Gov: NCT03706833) were prospective, multicenter, randomized, controlled trials intended to compare the two TEER devices in PMR and FMR, respectively. The one-year outcome of the CLASP-IID showed that the PASCAL system was safe, effective, and non-inferior to the MitraClip system in terms of clinical outcomes and echocardiographic results [22].

**Indirect Annuloplasty.** The only device with CE approval in this setting is the Carillon Mitral Contour System. It is positioned inside the coronary sinus (CS) or great cardiac vein (GCV) via jugular vein access, with general anesthesia usually required only when TEE guidance is used. The device consists of three components: (1) a proprietary implant intended for permanent placement inside CS/GCV, (2) a catheter-based delivery system, consisting of a curved delivery catheter, and (3) a sizing catheter. The implant is made of nitinol and titanium, manufactured with different lengths. It also has a distal and a proximal anchor, linked by a ribbon connector, and a proximal and distal crimp tube. The distal anchor is smaller and is placed inside the GCV, whereas the proximal one is larger and positioned near the CS ostium which is located near the posteromedial commissure of MV. The arc-shaped ribbon is designed to be deployed, tensioned, and secured inside the CS/GCV. Two previous trial, AMADEUS and TITAN I, showed improved clinical and echocardiographic parameters in patients who underwent the procedure, with a good safety profile [23,24]. However, cases of distal anchor slippage were documented with the first-generation device, leading to reengineering to reduce device failure, as shown in the TITAN II trial [25]. The subsequent randomized sham-control REDUCE FMR trial showed a significant reverse LV remodeling and a reduction in regurgitant volume [26]. These data were confirmed in a five-year period, which also denoted a benefit in terms of long-term survival [27]. Based on these promising results, a randomized trial is currently ongoing in Europe and the USA (ClinicalTrials.Gov: NCT03142152).

**Direct Annuloplasty.** The Cardioband Mitral System is a device used for direct annuloplasty TMVr through femoral vein access and TSP approach, under general anesthesia and TEE guidance. It consists of an implant system and three main accessories: (a) the Cardioband delivery system (CDS), (b) the implantable metal anchors and anchors delivery shafts, and (c) the size adjustment tool (SAT). The implant is a polyester sleeve, available in six sizes, with radiopaque markers; inside it, there is a metal wire connected to an adjusting spool that permits the shortening of the device at the end of procedure. The CDS is formed by an implant delivery system (IDS) and a transseptal steerable sheath (TSS); the IDS is formed by a steerable guide catheter (GC) and an implant catheter (IC) with the implant mounted on its distal tip. Between 12 and 17 stainless anchors are implanted using the delivery shaft. The SAT is used at the end of the procedure for cinching the device.

In a single-arm prospective multicenter experience of FMR treated with the Cardioband system, the device showed reasonable performance and safety [28]. However, additional data are necessary to further validate these findings. 

**Chordal Repair.** Chordal implantation is performed via transapical (TA) access in a mini-invasive procedure with two currently available devices: NeoChordDS1000 and HARPOON. These technologies allow for repair without annuloplasty and are considered viable alternatives primarily in cases of in MR without annular dilatation and/or LV remodeling (early-stage disease). The main difference from the surgical technique is that the distal extremity of the chorda is not fixed to the papillary muscle, but rather to a large pledget located at the apical access site, prompting the need for investigation into the long-term consequences of this approach.

The NeoChord system was the first TA chordal device available for use. The procedure is performed off-pump, under general anesthesia, through a left mini thoracotomy at the level of the intercostal space, which is suitable for TA access, and with TEE guidance [29]. Selecting the optimal entry site, usually postero-lateral, is crucial to avoid damage to the sub-valvular apparatus and ensure an equal distribution of tension among the neochordae. In some cases, alternative access sites and trajectories may be considered [30,31]. When the right position at the leaflet is reached, the jaws of the device are opened, and the device is withdrawn from LA to LV to grasp the leaflet. If other chordae are necessary, the device can be reloaded. The TACT trial showed that artificial chordae implantation is technically safe and feasible [32]. The ReChord trial (ClinicalTrials.Gov: NCT 02803957) is ongoing to evaluate the safety and efficacy of TA off-pump repair versus standard on-pump surgical techniques, and the AcChord trial (ClinicalTrials.Gov: NCT 04190602) is evaluating the five-year outcomes of patients treated with NeoChord DS1000 in a post-marketing setting. Moreover, a TS device (NeoChord NeXuS system) from the same company is under pre-clinical evaluation.

The HARPOON MVRS is a beating heart, off-pump, MVr system with a TA approach performed under general anesthesia and TEE guidance. The system consists of an introducer and a delivery system. When the tip of the delivery system is positioned under the leaflet, the double-helix coil knot is released by penetration through the leaflet tissue. The TRACER trial has explored the feasibility and safety of the device with positive results [33]. Another trial (RESTORE) is ongoing (ClinicalTrials.Gov: NCT04375332).

While early results are promising, the long-term durability and effectiveness of these procedures remain to be conclusively demonstrated. Ongoing research endeavors aim to address these critical aspects.

**TMVR.** Replacement is an alternative option for MR cases that are unsuitable for TMVr. The Tendyne system is the only CE marked device as of now; however, others are under investigation, including the Sapien M3 (Edwards Lifesciences), Intrepid (Medtronic), EVOQUE (Edwards Lifesciences), and others. TMVR can be carried out by a transfemoral or TA route, depending on the specific device, and is currently adopted in specific clinical settings, going from failed bioprosthesis [valve-in-valve (ViV)] to calcified valve [valve-in-mitral-annulus-calcification (Vi-MAC)] and failed prosthetic rings and bands [valve-in-ring (ViR)]. Each setting presents unique features, challenges, and outcomes [34]:

- *Vi-V*: This approach encompasses the implantation of a new bioprosthesis within a degenerated mitral bioprosthesis and is the most used out of the three. It is feasible via both TS and TA approaches. ViV procedures have demonstrated excellent results in terms of procedural (74%) and technical (94.4%) success rates, with a low incidence of post-procedural adverse events: 2.2% significant left ventricular outflow obstruction (LVOTO), less than 1% conversion to surgery, 3.3% significant paravalvular leak (PVL), and 6.2% 30-day mortality.
- *Vi-R*: This approach is based on the implantation of a bioprosthesis following a failed MV annuloplasty. The procedural success rate (57.4%) is lower compared to ViV procedures, with an increased risk of adverse events: 5% LVOTO, 12.6% PVL, 12% requiring a second valve implantation, and 9.9% 30-day mortality. The higher incidence of peri-procedural complications, along with significant residual MR, partly accounts for this elevated mortality rate. It is noteworthy that in patients previously subjected to MVr with an annuloplasty ring, TEER should be prioritized as the first option [35].
- *Vi-MAC*: This procedure poses significant technical challenges and is associated with a high-risk profile in the target population. Procedural success rates are comparatively lower (41.4%), with a higher incidence of complications: 8.6% conversion to surgery, 39.7% LVOTO, 6.9% valve embolization, 13% significant residual MR, 34.5% 30-day mortality, and over 60% one-year mortality. Due to these complexities and risks, Vi-MAC procedures are considered the most challenging among the various TMVR approaches. There are two ongoing trial that will provide additional information on the feasibility of TMVR in this setting, one with the Tendyne device (ClinicalTrials.Gov: NCT 0.539458) and the other with the Intrepid TMVR system (APOLLO, ClinicalTrials.Gov: NCT03242642).

Given the not infrequent absence of calcium at the level of mitral annulus (MA), different approaches have been proposed to ensure the proper sealing of the device, ranging from the D-shaped device profile to the use of apical tether, ventricular tabs, or anchors, in order to fix the device to the annulus, capturing the leaflets and chordae. Another technical strategy requires the realization of a two-step synching mechanism for device fitting.

The Tendyne valve is crafted for implantation in a beating heart, off-pump procedure using a TA approach.

**Figure 4:**
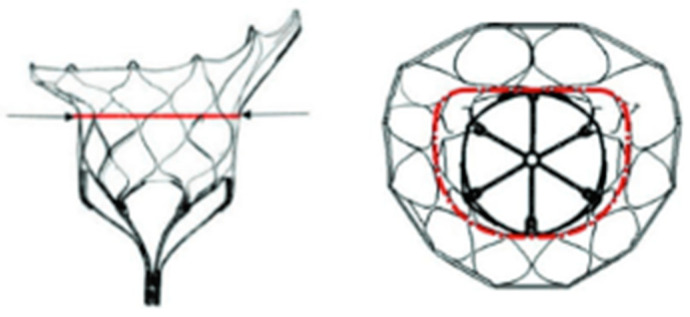
Tendyne valve seen from lateral (**left**) and overhead perspective (**right**). The black dark line corresponds to the valvular plane. The red line corresponds to the sealing portion of the device. Courtesy of Abbott.

Its design encompasses a self-expanded nitinol inner-frame, meticulously integrated with a porcine pericardial tissue tri-leaflet valve. A second self-expanding nitinol outer frame, fashioned with a D-shaped structure, is intricately linked to and envelops the inner frame. This outer frame is coated with porcine pericardium and a polyethylene terephthalate (PET) cuff, specifically engineered to enhance sealing with the native annulus and to mitigate the risk of paravalvular leaks PVLs (Figure 4).

The Tendyne valve offers two distinct models, each available in various sizes (refer to Table 1 for specifics). Supplementary components include the following: (a) an apical pad, available in small (S) and large (L) sizes, facilitating device anchoring at the apex; (b) a tether, which serves to interconnect and stabilize the valve with the pad; (c) a loading system; (d) a 36 F delivery system; (e) a pad positioning system; and (f) a 36 F retrieval system.

The valve’s design is meticulously structured to position it at the LA floor, with the tether and paddle mechanisms strategically positioned to impede prosthesis migration into the LA. Notably, the Tendyne device boasts full repositionability and retrievability, underscoring its versatility and precision in clinical application.

Its safety and efficacy have been explored in two trials, showing a success rate of 97.2% with a 5.5% 30-day mortality and a 1.8% stroke rate. The one-year mortality rate was 26%, mostly driven by cardiovascular mortality (85%). At one year, all patients showed a residual MR ≤ mild [36,37]. The ongoing SUMMIT trial (ClinicalTrials.Gov: NCT03433274) is going to evaluate TMVR with the Tendyne system versus TEER with MitraClip.

## 4. Pre-Procedural Evaluation

Echocardiography is the mainstay of MR diagnosis and quantification. It should be performed according to the latest guidelines [38,39]. The multiparametric approach is considered the reference for optimal MR grading, considering qualitative, semi-quantitative, and quantitative parameters. *Qualitative* parameters focus on structural aspects, such as MV morphology (prolapse, flail, perforation, ruptured papillary muscle, etc.), LV and LA size (normal or dilated), and Doppler signals, such as the color flow jet area (small/narrow/brief, large/eccentric/wall-impinging), the flow convergence (small/transient, large), and the continuous wave Doppler jet trace (faint/partial/parabolic, dense/triangular/holosystolic). *Semi-quantitative* parameters rely on vena contracta width (VCW < 0.3 cm, 0.3–0.6 cm, ≥0.7 cm), vena contracta area (VCA), pulmonary vein flow (systolic dominance, blunt, and reverse flow) and mitral inflow (A-wave dominant, E-wave dominant > 1.2 m/s); the last ones, together with LA and LV size, are more useful for assessing hemodynamic impact than for MR quantification. *Quantitative* parameters can help subclassify the moderate MR group and are represented by effective ROA (EROA), calculated by the PISA method, volumetric method, or pulsed-wave Doppler (PWD) method, and the regurgitant volume (RVol) and regurgitant fraction (RF).

**Figure 5:**
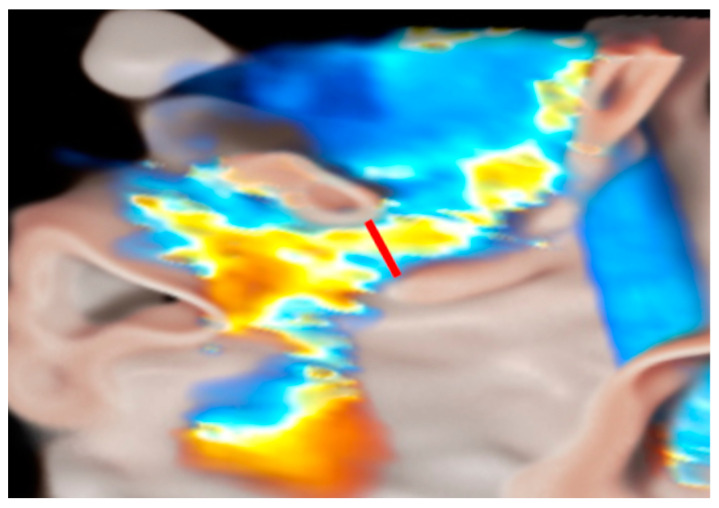
3D Color transillumination rendering of the LAX ME view of MR and vena contracta (red line). Abbreviations are as in the text.

It is important to be confident when faced with the peculiarity and pitfalls of these different parameters. For example, VCW corresponds to the narrowest portion of the MR jet, located immediately downstream from the anatomic regurgitant orifice (Figure 5). It is scarcely influenced by flow status, but it cannot be used in the presence of multiple jets and may overestimate MR when not holosystolic.

The VCA is measured with multiplanar reconstruction (MPR) from a 3D color dataset; this method offers the advantage of ensuring a precisely orthogonal cross-sectional area of the VC zone, free from dependence on flow-rate variations or any geometric assumption; its effectiveness is particularly pronounced in cases of non-circular shaped EROA [40]. Moreover, this method has been recently validated in a PMR population, suggesting an optimal cut-off of 0.45 cm^2^ for severe MR [41]. RVol, RF, and EROA can be measured with different time-consuming methods, of which the most widely used is the PISA one. Nevertheless, PISA may not be accurate with multiple or eccentric jets or with marked elliptically shaped EROA. The PWD method is valid only if at least one semilunar valve (aortic or pulmonary) has a regurgitation of no more than mild and there are no intracardiac shunts; however, even in this case, the risk of error is facilitated by the known nature of the equation used to calculate the cross-sectional area of the outflow tract.

Concerning volumetric methods, the primary limitation is the potential underestimation of left ventricular (LV) volume. However, this limitation can be partially mitigated through the utilization of contrast echocardiography or advanced 3D imaging tools [42,43].

### 4.1. Transcatether Edge-to-Edge Repair

**Figure 6:**
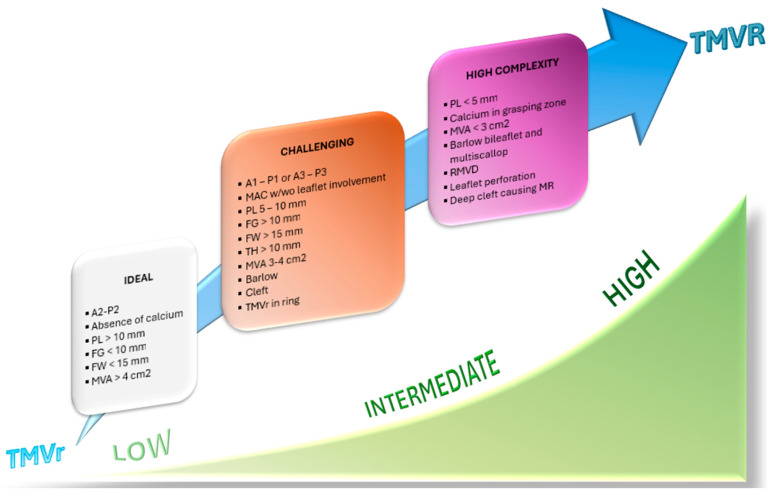
Volume experience (green hyperbola) and increasing anatomical difficulties (from white to purple box) determine the best therapeutic strategy. Easier anatomies suitable for repair can be treated even in centers with limited experience, but more difficult anatomies might require replacement strategies and complex repairs that should be performed in high-volume centers. PL, posterior leaflet length; FG, flail gap; FW, flail width, MVA, mitral valve area; MAC, mitral annulus calcification; TH, tenting height; TMVr, transcatheter mitral valve repair; TMVR, transcatheter mitral valve replacement; RMVD, rheumatic mitral valve disease.

Pre-procedural screening of patients proposed for TEER serves the crucial purpose of evaluating the mechanism of MR and confirming the absence of absolute anatomical contraindications in the procedure. Given the close correlation between volume experience and procedural success, the most complex anatomies should be referred to high-volume centers with specialized expertise in managing challenging cases (Figure 6). Therefore, it is currently more appropriate to classify anatomies into ideal, challenging, and high-complexity categories. Procedural interventions for challenging/high complexity anatomies should be performed in high-volume centers [44].

Anatomical evaluation of mitral disease should adhere to specific guidelines and necessitate standardized views [45]. The interatrial septum (IAS) should be carefully evaluated, as certain anatomies may pose challenges during TSP, such as small fossa ovalis, floppy septum, or atrial septal defect (ASD) closure device on site. In PMR, as previously observed, some criteria may predict successful grasping (flail gap < 10 mm, flail width < 15 mm) and the number of clips needed (flail width> or <10 mm) [46]. The PASCAL system may be the best choice in the presence of challenging anatomies [47]. In FMR, the selection criteria should focus on the identification of the COAPT-like criteria that better stratify the outcomes: FMR ≥ 3+, LV ejection fraction ≥ 20%, LV end-systolic diameter ≤ 70 mm, TAPSE ≥ 15 mm, TR grade < 3+, and systolic pulmonary artery pressure ≤ 70 mmHg [48].

### 4.2. Indirect and Direct Annuloplasty

The role of TEE in patients who are candidates for a Carillon device is limited to the evaluation of the MR mechanism, since the reference method to evaluate the CS anatomy and the correct size of the device is invasive venography. With the Cardioband system, it is necessary to give information on the MA morphology and size, the length of mitral leaflets, the course of the CS and left circumflex artery (LCA), the anatomy of the IAS, and the risk of LVOTO. In this context, research is also exploring the feasibility of radiation-free pre-procedural planning, particularly in patient subgroups where contrast multidetector-computed tomography (cMDCT) is deemed unsafe [49].

CT plays a pivotal role in the preoperative planning for transcatheter annuloplasty. The configuration of the mitral apparatus varies throughout the cardiac cycle; therefore, for the precise evaluation of the MV shape and dimensions, it is mandatory to utilize a retrospectively ECG-gated acquisition protocol [50,51]. This involves reconstructing CT datasets at intervals of 5% or 10% of the R-R interval, spanning from 0% to 100%. Tube voltage and tube current should be modulated to achieve the lowest possible radiation dose while maintaining good image quality, using 50–70 mL of contrast volume at a flow rate of 4–6 mL/s. 

Typically, the left side is made opaque for assessing the MV [52]. Introducing some contrast opacification on the right side is recommended to view the interatrial septum and aids in planning for transseptal access. CT coverage from the cranial to caudal areas should ensure complete visualization of the heart (usually from the carina to the diaphragm), which may be enlarged in cases of significant MVD disease [53]. Then, an additional chest scan without extra contrast is often conducted to examine the anatomy of the thoracic aorta for possible surgical planning and to determine the location of the apex relative to the ribs, especially for transapical access. Further helical scans of the abdomen and pelvis might also be necessary to assess the peripheral vasculature. An additional scan, about 60–90 s after the initial one, can improve the understanding of venous anatomy for trans-septal approaches. Thin submillimeter slices are reconstructed in the axial plane, and efforts are made to minimize radiation exposure by using the lowest feasible tube voltage and current while employing iterative reconstruction techniques. Scan triggering can be initiated using the bolus tracking or the bolus method [54].

CT allows the evaluation of the CS and LCA. Specifically, the LCA typically courses along the left atrio-ventricular (AV) groove between the posterior MA, the CS, and the great cardiac vein. In annuloplasty procedures, a very short distance between the MA and the LCA poses a risk of direct coronary damage or compression during device fixation. CT, with volume rendering and multiplanar reconstructions, enables the measurement of the distance between the MA and the LCA, aiding the operator in assessing the patient’s suitability for annuloplasty. Additionally, a wide angle between the CS and the MA may lead to inadequate force transmission to the MA, potentially resulting in procedure failure [50,51,55,56].

### 4.3. Chordal Repair

It is important to underline that not all anatomies are suitable for chordal repair. The ones expected to yield favorable outcomes are those with an isolated prolapse/flail of P2, exhibiting a single jet lesion, lacking annular and left ventricular (LV) remodeling, and possessing an appropriate length of the posterior leaflet compared to the antero-posterior (AP) mitral annulus diameter. TEE plays a fundamental role in the pre-procedural evaluation of these patients. It is essential for accurately classifying MV anatomy. In this regard, we can distinguish between several types, as follows:

- “Type A” (isolated central posterior prolapse/flail);
- “Type B” (posterior multi-scallop prolapse/flail);
- “Type C” (anterior or bileaflet prolapse/flail);
- “Type D” (para-commissural prolapse/flail or any significant annular or leaflet disease, e.g., calcification).

**Figure 7:**
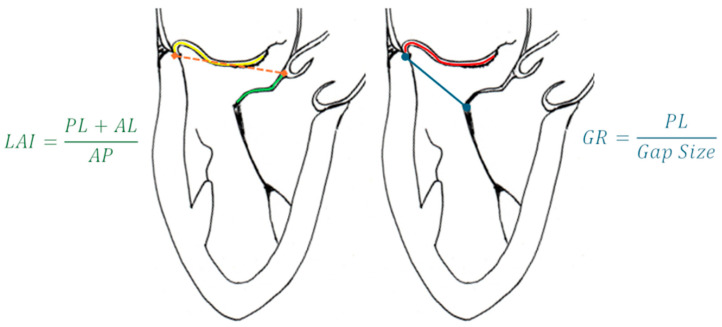
(**Left**) LAI; yellow line is the PL length, the green line is the AL length, and the orange dotted line is the AP diameter. (**Right**) Gap ratio; the red line is the PL length and the blue line the gap size. Abbreviations are as in the text.

Outcomes are more favorable in type A and B pathologies, whereas type D is generally considered unsuitable [57]. Mitral annulus (MA) dilatation is a critical determinant of procedural success. MA dilatation and the loss of saddle-shape can increase traction on the leaflets, potentially leading to procedural failures. Evaluating calcifications is also crucial, ensuring their absence in the target zone of chordal insertion. One of the most important echocardiographic parameters to assess is the leaflet-to-annulus index (LAI). This index, expressed as the ratio between the sum of anterior (AL) and posterior (PL) leaflet lengths divided by the antero-posterior (AP) diameter of the MV (AL + PL/AP), reflects the degree of leaflet–annulus mismatch. A cut-off value of ≥1.25 is associated with mild or less mitral regurgitation (MR) at one-year follow-up [58]. Another key parameter is the gap ratio, defined as the ratio between the PL length and the distance between the free edge of the anterior leaflet (AL) and the hinge point of the posterior leaflet (PL). A gap ratio value ≥ 1.5 correlates with improved outcomes, ensuring adequate leaflet coaptation [33] (Figure 7). These parameters serve as valuable tools in pre-procedural planning and risk stratification for chordal repair.

CT provides useful information about the pathology of mitral leaflets and MA size. It allows identification of prolapse with two- and three-chamber reconstructions that have high diagnostic accuracy compared to echocardiography [59]. In cases of mitral prolapse, small increments of reconstruction of the cardiac cycle (e.g., 5%) may be useful to allow for a detailed evaluation of the leaflets during systole. Instead, the MA dimension in patients with MR should be evaluated in the systolic phases (20–40%), where the largest dimension was found [60,61]. Furthermore, CT is also fundamental to evaluate MAC displaying the best trajectory line, choosing the optimal location of TA access [30,62].

### 4.4. TMVR

TMVR is a complex procedure with some characteristic complications (LVOTO, LCA compression, etc.), so the pre-procedural planning is undeniably crucial for accurate prediction of these risks and subsequent optimal planning.

TEE is necessary and can also potentially be used for pre-procedural screening of TMVR candidates [49]. However, cMDCT remains mandatory and is considered the reference method in this context. cMDCT provides detailed information regarding geometric remodeling of the MV, including precise measurements of leaflet heights and angles. Moreover, it offers superior visualization of the dynamic nature of the mitral annulus, which undergoes changes in functional anomalies of the MV. Understanding these anatomical nuances is crucial for proper device anchoring and can contribute to reducing fluoroscopic and procedural times [63,64]. By optimizing procedural planning and execution, cMDCT plays a pivotal role in enhancing the safety and efficacy of TMVR procedures. Furthermore, anomalies linked to with LV dilation and wall motion abnormalities (WMAs) can lead to the outward displacement of the papillary muscles, annular dilatation, and basal remodeling of the myocardium. This process can result in the formation of a characteristic “myocardial shelf”, which is identifiable on CT imaging [65]. Since the morphology of the myocardial shelf changes throughout the cardiac cycle, ensuring proper anchoring of the device to the infero-lateral basal myocardium requires dynamic sizing of the posterior myocardial shelf in both systole and diastole. Additionally, CT imaging enables assessment of the extent and severity of mitral annulus (MA) and leaflet calcifications. In most severe cases, such calcifications can pose significant risks, potentially rendering the procedure extremely hazardous. Thus, thorough evaluation of these factors via CT imaging is crucial for pre-procedural planning and ensuring the safety and efficacy of interventions, such as TMVR. CT also permits the differentiation of MAC from caseous calcification of the annulus, a rare variant, in which a large lesion occurs along the posterior annulus due to caseous transformation of the calcified ring material [66]. A dangerous complication of TMVR is LVOTO. The mechanisms of obstructions can be either fixed, as in the case of prosthetic obstruction, or dynamic, due to the anterior systolic movement of the anterior mitral leaflet secondary to the displacement of the prosthesis. CT with postprocessing software can evaluate some elements that predict LVOTO, such as the presence of a neo-LVOT area equal to or less than 1.7 cm^2^ [53]. CT allows optimization of the fluoroscopic angles used during the procedure to obtain the correct visualization and implantation of the prosthetic device to minimize complications during the deployment and positioning of the prosthesis [67,68].

## 5. Intra-Procedural Guidance

### 5.1. Transcatether Edge-to-Edge Repair

Before starting the procedure, it may be beneficial to reassess the severity of mitral regurgitation (MR). This allows for a more accurate comparison of the post-procedural outcome with the baseline condition, taking into account the common anesthesia-induced intra-procedural downgrading of MR [69]. Moreover, the re-evaluation of MR is fundamental to confirm the correct site of device implantation. In addition to MR quantification, a standardized protocol evaluation may also include PWD evaluation of pulmonary vein flow, the trend of which has a significant impact on patient’s outcomes [70]. The procedural steps of TEER are as follows:

(1) TSP and SGC advancement;
(2) Straddling and steering of the device;
(3) Orientation;
(4) Grasping;
(5) Final evaluation and release.

The trans-septal puncture (TSP) represents the most critical step, because a suboptimal crossing of IAS may negatively condition the complete procedure. The optimal site for the puncture is the postero-superior area of the fossa ovalis, identifiable through a bicaval view (defining supero-inferior orientation) and a short axis view at the aortic valve level (defining antero-posterior orientation). Tenting should be clearly visualized and the height from the MV plane must be measured in a modified mid-esophageal (ME) four-chamber view. In patients with primary mitral regurgitation (PMR), the acceptable height may range from 4.5 to 5 cm, whereas in functional mitral regurgitation (FMR), a height of 3.5 to 4.0 cm may be acceptable due to the more ventricular displacement of the coaptation zone. Additionally, medial pathology necessitates a higher transseptal puncture (TSP) to ensure adequate space for the system to bend back towards itself without crossing the MV plane. Conversely, lateral pathology may tolerate a lower TSP. Once the puncture has been performed, the guidewire is positioned inside the left upper pulmonary vein (LUPV). Subsequently, after removing the TSP system, the steerable guide catheter (SGC) with its dilator is advanced over the wire into the left atrium (LA). The echogenic knurled aspect of the dilator’s tip helps in its identification. Upon removal of the dilator, the double binary aspect of the SGC becomes visible. Although 3D imaging may seem attractive, 2D remains the preferred imaging modality to monitor this step due to its higher spatial and temporal resolution. At this point, the CDS is advanced inside the SGC to exit inside the LA.

**Figure 8:**
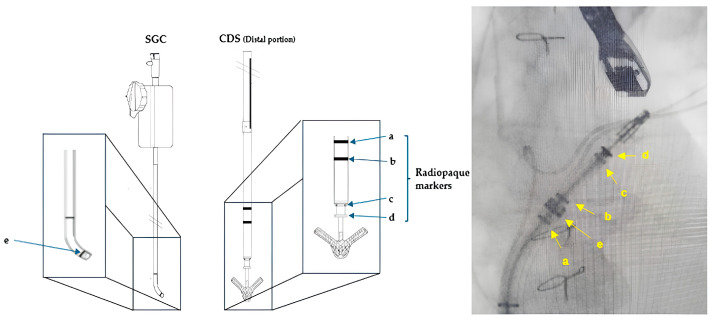
Straddling maneuver. Image on the left shows a focused representation of SGC and CDS, highlighting the radiopaque markers (adapted from Abbott). Image on the right enlightens the same markers on a fluoroscopic real-time view. (a, b, c, and d) are radiopaque markers on the distal portion of CDS. (e) is the radiopaque marker on the SGC. Abbreviations are as in the text.

The straddling is substantially a fluoroscopic maneuver, involving the alignment of the two markers on the CDS shaft with the marker on the tip of the SGC (Figure 8). This orientation points the system towards the LUPV, providing more maneuvering space for subsequent steering.

**Figure 9:**
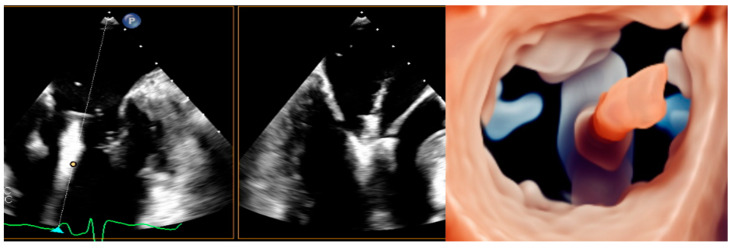
Steering step (**left** and **central** panel) consists of the descent of the device towards the MV plane. The orientation (**right** panel) is important to ensure a perpendicular alignment with the coaptation line. Abbreviations are as in the text.

After that, the CDS is steered towards the MV plane, aiming for a perpendicular alignment. The TEE standard views to monitor this step are the simultaneous bi-plane with medio-esophageal (ME) commissural view (standard plane) and the long-axis (LAX) view (derivate plane). Once the clip has arrived at the MV level, it is the time to align its arms perpendicularly to the coaptation line, using clockwise or counterclockwise rotation supported by a 3D en-face MV view (Figure 9).

**Figure 10:**
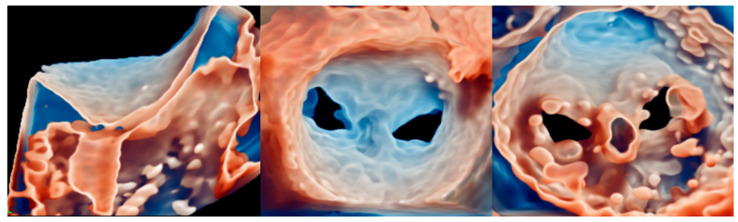
3D-zoomed view of the MV post-clip implantation. The images are visualized from lateral (**left**), en-face LA side (**central**), and LV side (**right**) views, using transillumination tools, with decreasing transparency for lower values. Abbreviations are as in the text.

Then the clip is advanced below the MV plane, with the arms partially closed. Subsequently, the arms are opened and the correct orientation is revaluated, as the system may rotate during the advancement (Figure 9). Subsequently, the opened arms are retracted towards the leaflets. Once the leaflets rest on the arms, the grippers are lowered to grasp them, ideally capturing a length of at least 5 mm. A good anatomical grasping is confirmed by the substantial reduction in leaflet mobility and the absence of a regurgitant jet inside the clip, confirming the total insertion of the leaflet’s edge inside the clip (Figure 10).

Indirect signs of mitral regurgitation (MR) reduction include a reduction in LA pressure and the appearance of spontaneous echo-contrast within the LA cavity. Additionally, it is crucial to assess the absence of stenosis, particularly when high flow velocity is observed on a color Doppler. These assessments aid in confirming the effectiveness of the procedure and ensure that no complications, such as stenosis, have arisen as a result of the intervention. This evaluation relies on a multiparametric approach, considering not only the transmitral gradient, but also pressure half-time (PHT), and, most importantly, the MV area (MVA) measured with multiplane reconstruction (MPR). It is crucial to note that each mitral orifice must be measured separately, as they lie in different planes. If the clip position is satisfactory, it is released. If necessary, additional clips can be implanted, ensuring a balance between MR downgrading and MVA reduction. The subsequent clips are usually advanced with arms closed to minimize the risk of damage to the previously implanted one. When all the clips have been placed, the CDS is retracted inside the SGC and is important to carefully monitor this step, because the sharp tip of the shaft may damage the LA wall. With the CDS inside the SGC, the system is then retracted and removed.

The PASCAL system follows similar procedural steps to the MitraClip system, with one notable exception: the “parallelism test”. This test is performed after TSP and used to ensure that the guide sheath tip flexes parallel to the MV plane. Monitoring this alignment can be achieved with a 3D view of the interatrial septum (IAS) from the left atrial (LA) perspective, with the MV positioned at six o’clock. 

While fluoroscopy is traditionally considered a complementary modality, particularly for interventionalists who may initially rely more on fluoroscopic projections during the learning process, it also provides valuable support for interventional echocardiographers (IE) in maintaining procedural control. However, recent data suggest the potential feasibility of a radiation-free procedures. Additionally, in cases of complex mitral anatomy and challenging transesophageal echocardiography (TEE) imaging, the use of fluoroscopic–TEE fusion imaging may become necessary to enhance procedural guidance and accuracy [71,72].

### 5.2. Indirect Annuloplasty

**Figure 11:**
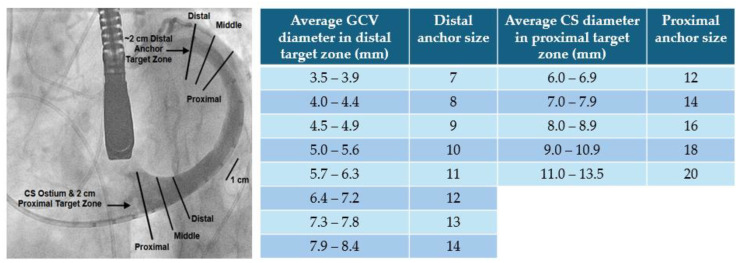
CS venography with sizing measurements (**left**, courtesy of Cardiac Dimensions) and Carillon XE2 sizing chart (**right**). Note that the distal anchor must be placed in a target zone with a minimum diameter of 3.5 mm, whereas the proximal anchor is in a target zone with a maximum diameter of 13.5 mm. Abbreviations are as in the text.

The first procedural step consists of coronary artery angiography to evaluate possible disease, anatomy and flow of coronary arteries and to identify the CS ostium through the venous phase after contrast injection. Subsequently, cannulation of CS is conducted via right internal jugular vein access using the delivery catheter and a diagnostic catheter (i.e., a multipurpose 6F or 7F) in a telescopic manner, with the last inside the first and over a guidewire, positioned up to the anterior interventricular vein (AIV). The diagnostic catheter is advanced until it reaches the GCV/AIV junction and, after that, the delivery catheter is advanced over the diagnostic one to reach the same position. Then both the diagnostic catheter and guidewire are removed, and the sizing catheter is advanced inside the delivery catheter. A second coronary artery injection in right anterior oblique (RAO) caudal projection is performed to evaluate the relationship of the LCA with CS/GCV, identified by the presence of the delivery catheter. At this point, a venogram of the coronary venous system is performed with a retrograde injection. Both angiographies are also repeated in left anterior oblique (LAO) caudal projection. For the sizing of the device, an average of three measurements both in the distal and proximal target zones is used (Figure 11).

The subsequent step involves the insertion of the device, followed by the release of the distal anchor and the retraction of the delivery catheter, which is then pushed forward to facilitate the expansion of the anchor. During this step, a coronary angiography injection is conducted to confirm the flow of the left coronary artery (LCA).

Following this, manual traction is applied to plicate the peri-annular tissue and cinch the annulus, after which the proximal anchor is released. Another coronary angiography is performed to ensure the absence of extrinsic compression of the device on the LCA.

At the conclusion of the procedure, if the patient is intubated, TEE can be utilized to assess the outcome. Alternatively, if the patient is only sedated, a transthoracic approach may suffice for evaluation.

### 5.3. Direct Annuloplasty

TEE has a central role in the Cardioband procedure in order to allow the correct implantation of all anchors, which is necessary to achieve procedural success.

The site of TSP is evaluated in pre-procedural planning with TEE/CT analysis and must be located above the postero-medial commissure at a >3.5 cm height from MV plane. In this case, 3DTEE may be extremely useful, allowing a direct visualization of the commissure and the IAS in a single view (3D LA perspective “en-face” IAS view); at the rotation of the 3D dataset from an overhead perspective allows a better visualization of the tenting site.

After the TSP, the TSS and the IDS are advanced into LA. The first site of implantation is the antero-lateral commissure, near the left trigone, where the first anchor must be released. This zone is identified through fluoroscopy (the LAO and RAO view pre-identified by CT) and TEE guidance, with both the 2D and 3D view.

This step is crucial, as an incorrect implantation of the first anchor may jeopardize the success of the procedure. Subsequently, the second and third anchor are positioned near the first one, usually in the first 12 mm, to ensure a better stability of the device; the others are then implanted every 8 mm until the postero-medial commissure (right trigone) has been reached. Every anchor is positioned under fluoroscopy and TEE guidance, using both 3D en-face and MPR, because it is necessary to maintain an entry angle of 45° from the LA cavity towards the annulus and an angle of penetration of 90° along the implantation line. After the release of the last anchor, the IDS is withdrawn, and the SAT is inserted into the TSS. The SAT allows the shrinkage of the device and annulus, resulting in the reduction in MR grade.

### 5.4. Chordal Repair

During these procedures, which are conducted under general anesthesia and TEE guidance, TEE is the sole imaging modality that is necessary. After performing a left mini-thoracotomy, the apical access site is identified using bi-plane ME commissural (main plane) and ME-LAX views (derived plane) with the “finger poking” maneuver. Ensuring the correct site of entry is critical to preserve the integrity of the sub-valvular apparatus.

With the NeoChord system, the device is advanced to attain a position between the leaflets. Subsequently, its jaws are opened and then closed to grasp the leaflet, simultaneously releasing a loop suture and a girth hitch knot at the level of the mitral leaflet. After the first neochord release, the device can be reloaded, and additional sutures (typically up to 3–4) can be placed. The length of the neochordae can be adjusted to achieve the best anatomical result and optimal reduction in MR grade. The other end of each neochord is anchored to a pledget at the access site in the LV.

With the HARPOON device, TEE is used to confirm the correct position of the delivery system and the contact of the end effector with the leaflet; at this point the plunger is released to deploy a double-helical knot of e-polytetrafluoroethylene (ePTFE). If more than one ePTFE chord is required, the rule is to start lateral and then go medial.

### 5.5. TMVR

The only CE-marked device for TMVR is the Tendyne, so our focus will be on this device. The procedure is performed under general anesthesia, with hemodynamic monitoring in an operating room. The apex is reached with a left mini-thoracotomy, and a lateral puncture is performed to insert a J-tip guidewire from the LV to the LA. The correct site of puncture is chosen to be perpendicular to the MV plane and to be safety towards the subvalvular apparatus and the “finger poking” maneuver is used, as in chordal repair techniques. The procedure is monitored with bi-plane ME commissural and LAX views. A 34 F sheath is advanced over the wire into the LA and the implant device is then inserted into the sheath and progressively extruded until it expands up to 85% of its final size. This step is monitored with an LA perspective en-face of the MV view using 3DTEE. The correct anatomical position is chosen in order to have the higher cuff leaning against the mitro-aortic curtain area. The device is then withdrawn towards the ventricle, gaining an intra-annular sealing. The valve is linked to the pad with the “tether”, and the pad is fixed at the level of LV access. The tension of the tether is adjusted to ensure an optimal position of the device.

## 6. Post-Procedural Evaluation

Echocardiography plays a crucial role in the post-procedural evaluation of TMVI, as it is able to assess procedural success and check for potential complications. Procedural success evaluation relies on the accurate knowledge of the device-specific procedural steps and mechanism of function, also remembering that correct evaluation of residual regurgitation requires the integration of multiple parameters [73,74]. 

Regarding the complications, excluding those that can occur after numerous percutaneous procedures (e.g., bleedings, access site damage, contrast-induced nephropathy, pericardial effusion due to damage of cardiac wall, etc.), the focus will be moved onto the most common device-related complications.

### 6.1. TEER

TEER is generally considered a safe procedure; however, it could be associated with some serious complications that need to be identified promptly. Some examples include cardiac tamponade, potentially correlated to suboptimal TSP, acute deterioration of LV dysfunction due to the afterload mismatch, or unexpected worsening of MR; all these complications may lead to acute cardiogenic shock.

A feared complication is MR aggravation, which can be potentially related to acute LV dysfunction, leaflet or chordal damage, loss of leaflet insertion (LLI), and partial leaflet detachment (PLD). While some of these factors may be predictable, others, such as iatrogenic leaflet or chordal damage, are considered avoidable complications that may result from technical errors during the procedure, such as multiple grasping attempts or navigating into non-chordal free areas [75]. 

Although the frequency of these complications may be relatively low, accurate patient selection is crucial to minimize their occurrence. Post-procedural evaluation should include a multiparametric approach to check for complications, such as mitral stenosis. This evaluation typically begins with the assessment of the transmitral gradient and pressure half-time (PHT) and ends with the measurement of MVA using MPR techniques. MVA measurement with MPR is particularly valuable as it provides highly predictive information regarding adverse outcomes associated with mitral stenosis following TEER procedures [76]. Iatrogenic atrial septal defects (ASDs) resulting from TEER procedures are typically considered to have negligible clinical significance, particularly during long-term follow-up [77,78]. 

### 6.2. Annuloplasty Devices

When utilizing annuloplasty devices, particular attention must be given to the risk of left circumflex artery (LCA) injury, which manifests as wall motion abnormalities (WMAs) within the LCA territory. In the context of Cardioband procedures, particularly in during initial experiences, there is the additional concern of anchor disengagement. This complication may occur during the cinching step or following the pull test, potentially leading to partial device detachment, especially at the lateral commissure or within the P2 area. Additionally, the proximity of the medial commissure to the atrioventricular (AV) node necessitates consideration of the risk of AV conduction disorders, a concern applicable to all direct annuloplasty procedures.

### 6.3. Chordal Repair

The specific complications associated with chordal repair techniques are comparable to those encountered in transcatheter edge-to-edge repair (TEER), as both involve a leaflet approach, albeit from different access points (e.g., apex). A primary concern in chordal repair is the potential detachment of the chord, leading to procedure failure. However, with careful patient selection, such occurrences are typically rare and can be considered anecdotal.

### 6.4. TMVR

When discussing TMVR, it is essential to distinguish between peri-procedural (short-term) and post-procedural (long-term) complications. Specifically focusing on device-related issues, critical aspects to assess include the risk of LVOTO, valve embolization or migration, valve thrombosis or dysfunction, the emergence of paravalvular leaks, and the atrio-ventricular (AV) groove injury [79].

LVOTO is a potentially life-threatening complication that necessitates prediction through pre-procedural evaluation, employing different modalities [49,54]. In this context, it is advisable to adopt strategies to prevent LVOTO, such as transcatheter laceration and translocation of the anterior mitral leaflet (e.g., LAMPOON and BATMAN techniques), or septal ablation using radiofrequency techniques or alcohol. These methods are also utilized to treat post-procedural LVOTO.

Valve migration or embolization can occur as an early complication due to suboptimal positioning of the device. Conversely, it may manifest as a delayed complication following an episode of prosthetic valve endocarditis or as a consequence of an undersized device experiencing delayed migration due to elevated left ventricular pressure, especially in the absence of extensive peri-device calcification [80].

Valve thrombosis or dysfunction and paravalvular leaks are complications that can occur with any prosthesis, and their pathophysiology closely resembles that of surgically implanted valves. Similarly, the methods for imaging detection and evaluation are akin to those used for surgical prostheses.

The AV groove injury and rupture are frightening complications, usually facilitated by the presence of a small left ventricle and an oversized device.

## 7. Emerging Techniques and Potential Future Directions

**Figure 12:**
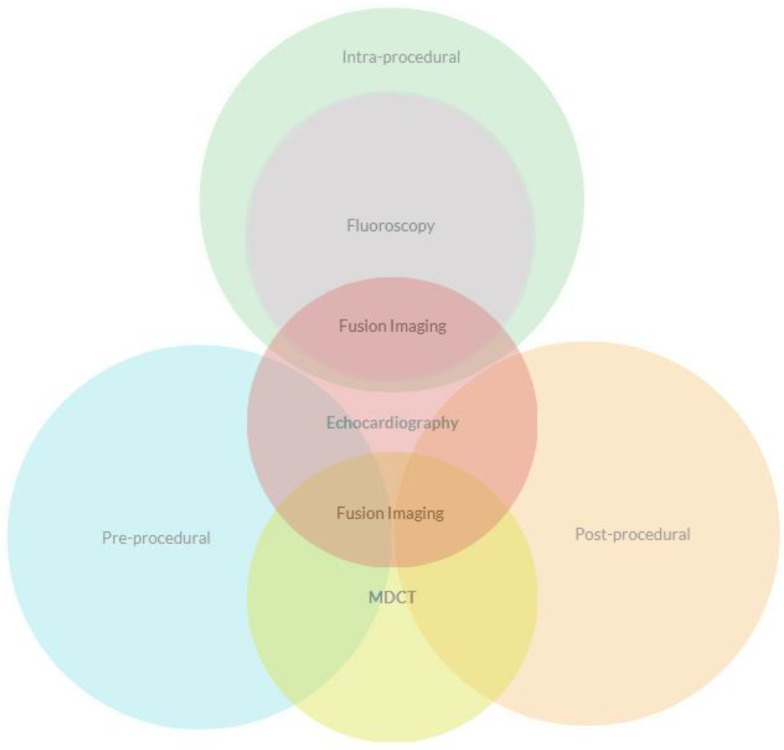
Relationship of imaging methods during the procedural steps of TMVI.

Echocardiography represents the cornerstone of imaging dedicated to TMVI. Despite its linchpin role in TEER, covering all the steps from pre- to intra- and post-procedural evaluation, it still needs the irreplaceable support of MDCT and/or fluoroscopy in many cases (Figure 12).

Moreover, in some patients, there may be a suboptimal TEE acoustic window due to the supine position, lipomatosus interatrial septum, or acoustic shadowing from prostheses or chest structure. In this setting, the intracardiac echocardiography (ICE) may display all its advantages and completely substitute TEE for procedural guidance. ICE may provide real-time images with a high near-field resolution, 3D imaging and multiplanar reconstructions, allowing for anesthesia-free intra-procedural guidance [81,82]. Currently, the main limitation is represented by the single-use device and the lack of widespread experience with device manipulation.

Furthermore, cardiovascular magnetic resonance (CMR) can play a crucial role in diagnosing MR in the pre-procedural phase. Indeed, it is considered the gold standard for evaluating biventricular volume and function and it offers superior reproducibility and prognostic value for primary MR compared to TTE [83]. CMR can precisely measure the effective forward LV ejection fraction, even in cases of severe MR, by calculating the aortic forward flow volume relative to the LV end-diastolic volume [84]. Moreover, CMR can detect myocardial fibrosis, using late gadolinium enhancement (LGE) sequences, which has been shown to correlate with MR severity and LV dilation. Additionally, CMR provides a reliable evaluation of RV ejection fraction, which typically decreases with increased MR severity [85,86].

The sharp and unceasing progress in the field of structural heart disease (SHD), as well as the evolution in imaging techniques, has pushed us to turn our attention to the chance to take advantage of these innovations to resolve different problems.

**Radiation exposure (RAE).** One of the main issues in the field of SHD is the recent evidence of the considerable risk of RAE, underlining the imperative to mitigate this risk, especially for interventional echocardiographers (IE) [87]. In addressing this challenge, various approaches may be considered, ranging from the implementation of dedicated structural suite designs and shielding techniques to the development of tailored dose reduction programs, all with the ultimate goal of advancing towards minimally invasive and non-fluoroscopic procedures [71,88,89]. Efforts to reduce fluoroscopy time are paramount; potential strategies include implementing focused protocols or leveraging advancements in artificial intelligence (AI). AI holds promise in predicting the optimal fluoroscopic view from CT datasets through virtual fluoroscopy tools, as well as in facilitating fusion imaging for enhanced procedural guidance [90,91].

**Education.** Another aspect to consider is that the IE plays a pivotal role in the heart team and is crucial at all stages of valvular heart disease management. Indeed, fulfilling this role requires comprehensive education encompassing clinical, anatomical, imaging, and surgical knowledge. However, the limited availability of dedicated structural imaging fellowships poses a challenge, especially if formal certification processes require supervision by certified faculty [92]. In light of the increasing prevalence of transcatheter techniques, it is imperative to take steps to prevent the emergence of a class of IEs who are primarily self-educated. AI and virtual reality (VR) technologies can play a crucial role in addressing this issue by connecting individuals regardless of spatial and temporal constraints. These technologies facilitate real-time interactions in a 3D space with multimodal imaging capabilities, offering the potential for a comprehensive and immersive learning experience [93]. Indeed, this capability theoretically enables the sharing of numerous imaging cases, conferring benefits for both operator learning and patient management.

**Cooperation.** Knowledge of the procedural steps of the different devices and their different components, and a correct integration of echocardiographic and fluoroscopic imaging, is of substantial importance for procedural success. The IE should possess the ability to swiftly transition between different views, often resorting to off-axis projections if necessary. This skill necessitates a profound understanding of the three-dimensional orientation of anatomical structures, as well as the capability to anticipate the movements of the interventionalist, alerting them in cases of dangerous positions or trajectories. Therefore, these two operators must exhibit the aptitude to work together in a collaborative and efficient manner. Fusion imaging serves as a point of connection, enabling both operators to overcome the limitations of a single imaging modality. It facilitates improved visualization of soft tissue in fluoroscopy and radiopaque materials, guidewires, and delivery systems in echocardiography. Additionally, fusion imaging aids in reducing fluoroscopy time, thereby enhancing safety, accuracy, and procedural effectiveness [67,90,94].

**Prevision.** The utilization of AI, as proposed by recent studies and with the aid of suitable tools, holds the potential to predict the outcomes of MVI in the near future. This prospect carries significant implications, including the ability to customize procedures more precisely, optimize device and patient selection, and impartially assess anatomical variables [95,96].

## 8. Conclusions

Transcatheter mitral valve interventions (TMVIs) have experienced significant growth in recent decades, emerging as a well-established alternative for treating mitral regurgitation (MR) in patients who are ineligible for or at too high a risk for surgery. Several devices have been developed, with some receiving regulatory approval, such as CE mark and/or FDA approval, while others are undergoing evaluation. While the MitraClip system is the most widely utilized, several other devices are emerging as potential therapeutic options, particularly for patients with complex anatomies.

Cardiac imaging assumes a central role in assessing patients undergoing these procedures, requiring skills and knowledge distinct from those of basic echocardiography. This underscores the necessity for a specialized imaging sub-specialty with dedicated expertise [45,97]. Multimodal imaging is crucial throughout the TMVI process, aiding in preoperative evaluation of patient anatomy, predicting potential complications, guiding intraoperative interventions through hybrid imaging techniques, and promptly detecting postoperative complications.

The progress in the interventional and imaging fields is inherently intertwined. Technological innovations can yield devices and procedures that are increasingly suitable and effective. Simultaneously, Advancements in imaging technology empower healthcare professionals to achieve earlier diagnoses, preemptively identifying conditions before symptoms arise. This proactive approach significantly enhances prognoses by enabling timely interventions and treatments. Furthermore, ongoing research into multimodal imaging, machine learning, and virtual reality may enable us to harness the remarkable capabilities of artificial intelligence (AI) for processing, analysis, and data elaboration. This facilitates the utilization of these technologies not only to enhance the quality of disease treatment, but also for educational purposes.

## Figures and Tables

**Table 1:** Sizing chart of the Tendyne valve. AP, antero-posterior diameter. IC, intercommissural diameter. EOA, effective orifice area.

Model	Number	AP (mm)	IC (mm)	Perimeter (mm)	EOA (cm^2^)
**SP**	33S	32.5	43.5	130	3.0
	33M	32.5	46.5	136	
	33L	32.5	50.5	144	
	35M	34.5	48.5	144	
	37S	36.5	46.5	144	
	37L	36.5	52.5	156	
	39M	38.5	50.5	156	
	41S	40.5	47.5	154	
**LP**	29S	29.0	42.5	119	2.2
	29L	29.0	47.5	129	
	33S	32.5	43.5	130	
	35M	34.5	48.5	144	
	37M	36.5	49.5	150	

## Abbreviations

AI	Artificial intelligence
ASD	Atrial septal defect
AV	Atrio-ventricular
cMDCT	Contrast multidetector-computed tomography
CS	Coronary sinus
FMR	Functional mitral regurgitation
GCV	Great cardiac vein
IAS	Interatrial septum
IE	Interventional echocardiographer
LA	Left atrium, left atrial
LCA	Left circumflex artery
LV	Left ventricle, left ventricular
LVOT	Left ventricular outflow
LVOTO	Left ventricular outflow obstruction
MA	Mitral annulus
MR	Mitral regurgitation
PMR	Primary mitral regurgitation
PVL	Paravalvular leak
PWD	Pulsed-wave Doppler
RAE	Radiation exposure
SHD	Structural heart disease
TEE	Transesophageal echocardiography
TEER	Transcatheter edge-to-edge repair
TMVI	Transcatheter mitral valve intervention
TMVr	Transcatheter mitral valve repair
TMVR	Transcatheter mitral valve replacement
TR	Tricuspid regurgitation
Vi-MAC	Valve in MAC
Vi-R	Valve in ring
Vi-V	Valve in valve
VR	Virtual reality
WMAs	Wall motion abnormalities
